# Combination of Intravenous Ondansetron and Intravenous Glycopyrrolate Versus Intravenous Ondansetron and Intramuscular Glycopyrrolate for Reduction of Post-spinal Hypotension in Cesaraen Section: A Randomized Double-Blinded Study

**DOI:** 10.7759/cureus.72436

**Published:** 2024-10-26

**Authors:** Sandip Baheti, Sharan Muruganantham

**Affiliations:** 1 Anaesthesiology, Dr. D Y Patil Medical College, Hospital and Research Centre, Dr. D Y Patil Vidyapeeth (Deemed to be University), Pune, IND

**Keywords:** bezold-jarisch reflex, caesarean section, glycopyrrolate, ondansetron, post spinal hypotension

## Abstract

Background: In parturients, post-spinal hypotension is common due to loss of sympathetic tone. Compression of the inferior vena cava by the gravid uterus further aggravates it. Various pharmacologic and non-pharmacologic techniques are used to reduce the severity of hypotension. Ondansetron, a selective 5-hydroxytryptamine receptor antagonist, nullifies the Bezold Jarisch reflex response to spinal anaesthesia and glycopyrrolate, an anti-cholinergic, has been used to prevent post-spinal hypotension. We estimated the efficacy of intravenous (IV) glycopyrrolate plus IV ondansetron versus intramuscular (IM) glycopyrrolate plus IV ondansetron to reduce post-spinal hypotension in caesarean section. We compared hypotension episodes and the total ephedrine dose required to treat hypotension among the groups.

Methods: Ninety parturients undergoing elective and emergency caesarean section under spinal anaesthesia were randomly assigned into three groups of 30 each. Group A received prophylactic IV glycopyrrolate 0.2mg plus IV ondansetron 4mg, Group B received IM glycopyrrolate 0.2mg plus IV ondansetron 4mg and Control Group C received 10ml of normal saline plus IV ondansetron 4mg. The total ephedrine dose required to treat hypotension (primary objective), incidence of hypotension and maternal heart rate variations were analysed.

Results: Group B required significantly less vasopressor as compared to Group C (6.4286mg±1.6036 vs 8.7mg±3.6288, p=0.0420). There is no statistically significant difference between Group A and Group C for vasopressor requirement (7.8750mg±2.8723 vs 8.7mg±3.6288, p=0.5425). Maternal heart rate in Group A was higher at the eighth minute compared to Group B and Group C (100.2±21.3 vs 95.3±14.4 vs 86.2±19.0, p=0.015). Incidence of hypotension was same in all the groups (46.7% vs 46.7% vs 51.1%, p=0.491) and complications among groups were comparable.

Conclusion: Prophylactic use of IM glycopyrrolate plus IV ondansetron decreases total vasopressor required to treat hypotension but does not decrease the incidence of hypotension. Prophylactic use of IV glycopyrrolate increases maternal heart rate.

## Introduction

For elective and emergency caesarean section, the most common regional technique is spinal anesthesia [1]. Subarachnoid block-induced sympatholysis causes vasodilatation which leads to maternal hypotension, bradycardia and compromise in uterine blood flow, leading to foetal acidosis which may be detrimental to parturient and baby. Prevention of post-spinal hypotension during caesarean section has been a research study for more than 50 years [2] and the efficient strategy to achieve hemodynamic stability remains demanding [3].

Various pharmacological approaches like preloading with crystalloids and colloids, use of vasopressors like ephedrine [4], phenylephrine [5] and mephanteramine are in use. None of the non-pharmacological methods like left uterine displacement, wrapping and compression stockings of lower limbs [6] has been claimed to be useful. According to some studies preloading with crystalloids is not so much effective because volume preloading is not likely to prevent the spinal anaesthesia-induced decreased systemic vascular resistance [7,8]. Vasopressors like phenylephrine or ephedrine drip are used intravenously but their position has been provoked because of raised heart rate, tachyphylaxis and foetal acidosis [9-12]. Phenylephrine provides better fetal acid base values compared to ephedrine while maintaining similar efficacy in blood pressure control [13]; however, it is associated with reflex bradycardia. Additionally, serial dilution for intravenous administration can be a potential source of error.

From the current studies, ondansetron, a selective 5-hydroxytryptamine receptor antagonist, nullifies the Bezold Jarisch reflex response to spinal anaesthesia [14] and has an advantage in preventing bradycardia and hypotension [15]. Ondansetron is administered intravenously which has better onset of action and bioavailability than intramuscular. Glycopyrrolate, an anticholinergic, has been used to prevent post-spinal hypotension [16]. It has a chronotropic effect through the anti-muscarinic activity [17]. Various studies showed that intravenous glycopyrrolate is useful in prevention of post-spinal hypotension during caesarean section, but was associated with unanticipated maternal tachycardia [18] which can get exaggerated when oxytocin is given after delivery of the baby.

Recent studies indicate that intravenous (IV) ondansetron and glycopyrrolate when given pre-emptive to spinal anaesthesia decreases vasopressor demand in treating hypotension. Efficacy of combination of intramuscular (IM) glycopyrrolate plus IV ondansetron upon hypotension during spinal anaesthesia for lower segment cesarian section (LSCS) is not studied yet and combination of IV ondansetron and glycopyrrolate with the preferred route is not studied sufficiently. So the objective of our study was to compare the efficacy of combination of prophylactic IV glycopyrrolate plus IV ondansetron versus IM glycopyrrolate plus IV ondansetron for reduction of post-spinal hypotension during caesarean sections [7-9].

## Materials and methods

This prospective randomized double-blind study was conducted at the Department of Anaestheiology, Dr. D. Y. Patil Medical Hospital and Research Center, Pimpri, Pune, from June 2023 to December 2023 after Institutional Ethics Committee approval (IESC/PGS/2022/159) and Clinical Trials Registry India (CTRI) registration (CTRI/2023/05/053038). Written informed consent was obtained from all patients who consented to participate. Double blinding was followed throughout the study. Sealed opaque envelopes were used to conceal group allocation.

Assuming the effect size of 0.35 with an alpha error of 0.05 with power of 80%, minimum sample size calculated is 84 with 28 in each group. However we included 30 participants in each group with a total of 90 participants in this study. The sample size was calculated by assessing the incidence of hypotension and calculating rescue vasopressor used from previous articles by Manem et al. [19] and Vadhanan et al. [20]. Statistical package used was G03.1.9.7.

Ninety pregnant females posted for LSCS were divided into Group A, Group B, Group C. Group A (n=30) had IV glycopyrrolate 0.2mg plus IV ondansetron 4mg five minutes before spinal anaesthesia. Group B (n=30) had IM glycopyrrolate 0.2mg 15 minutes before spinal anaesthesia and IV ondansetron 4mg five minutes before spinal anaesthesia. Group C (n=30) had IV ondansetron 4mg and IV normal saline five minutes before spinal anaesthesia.

Selection was done using a computer-generated random number table. Pre-anesthetic checkup was done. All standard monitors (non-invasive blood pressure (NIBP), pulse oximeter, three-lead ECG) were connected and baseline values of parameters (blood pressure, heart rate, saturation and respiratory rate) were recorded. After securing 18G wide bore cannula, parturients were preloaded with 500mL of Ringer lactate and IV pantoprazole 40mg was given. All the intravenous drugs under study were diluted in normal saline to total amount of 10mL and intramuscular drug (glycopyrrolate 0.2mg) undiluted were prepared by another anaesthesiologist not involved in monitoring and analysis. Study drugs were given by anaesthesiologist who was blinded to the medications under study.

Spinal anaesthesia was given in a sitting position, under all aseptic precautions, at L3-L4 intervertebral space, using 26G Quincky spinal needle with 2mL of injection bupivacaine heavy 0.5% in a single prick. A pelvic wedge was placed over right buttock to prevent aortocaval compression. Supplemental oxygen was given via face mask at 5L/min. Bromage scale [19] was used to evaluate motor block every two minutes until complete motor block was achieved, which is mentioned as shown in Table 1. Surgery was started after sensory level of T6-T7 block which was confirmed by pin prick.

**Table 1:** Bromage Score

Grade	Criteria	Degree of Block
I	Free movement of legs and feet	Nil (0%)
II	Just able to flex knees with free movement of feet	Partial (33%)
III	Unable to flex knees, but with free movement of feet	Almost complete (66%)
IV	Unable to move legs or feet	Complete (100%)

Pulse, blood pressure, oxygen saturation, were recorded every two minutes after giving spinal anaesthesia for 15 minutes. Injection oxytocin 20IU was given through drip after delivery of the shoulder of baby. Hypotension is defined as fall in systolic blood pressure of less than 20% of the baseline value recorded. Blood pressure below 100mmHg was treated with injection ephedrine 6mg intravenously bolus and total ephedrine dose required was recorded. Bradycardia is defined as heart rate less than 50 beats per minute. Symptomatic bradycardia was treated with injection atropine 0.6mg intravenously. Shivering was treated with injection tramadol 50mg intravenously.

At the end of the study, all data were compiled and analyzed statistically with IBM SPSS Statistics for Windows, version 29.0 (IBM Corp., Armonk, NY, USA). Mann-Whitney U test applied for continuous data analysis. The Chi-square test was used for categorical data analysis. To identify significant differences in the multivariate analysis, a one-way ANOVA followed by Tukey’s post-hoc test was used. In all the statistical methods mentioned above, a probability value of ≤0.05 is considered a significant level.

## Results

**Figure 1:**
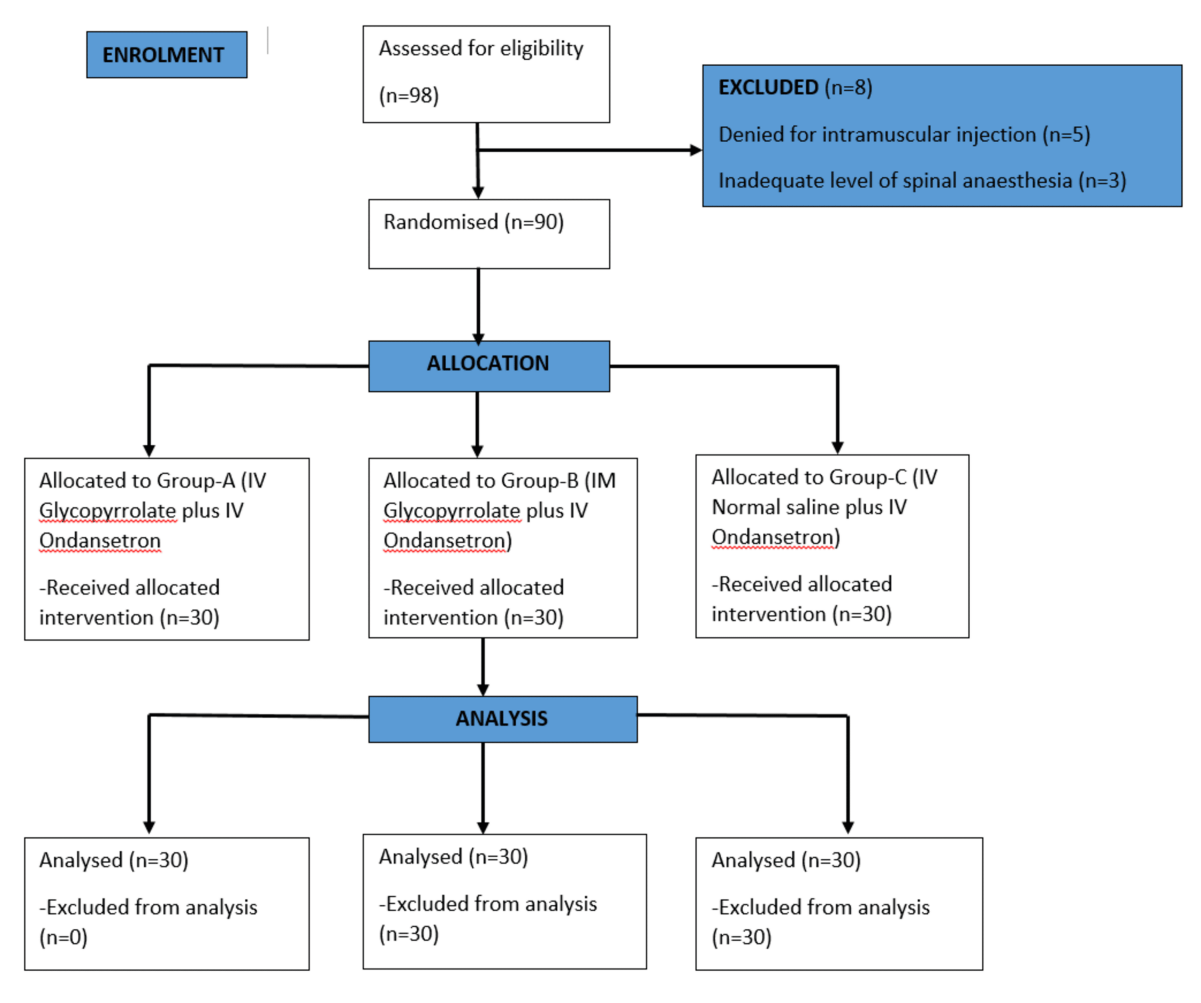
Consolidated Standards of Reporting Trials (CONSORT) Flow Chart

Ninety parturients of ages between 20-40 years scheduled for elective and emergency caesarean section American Society of Anesthesiologists (ASA)-I and ASA-II were included in study. Individuals with pregnancy-related comorbidities, systemic disorders, coagulopathies, anaemia, heart rate more than 100 per minute, hypersensitivity to glycopyrrolate and ondansetron were excluded. The Consolidated Standards of Reporting Trials (CONSORT) chart of the study is shown in Figure 1.

It compares three groups (A, B, and C) based on age, height, weight, and Body Mass Index (BMI), with all values presented as means with standard deviations. There are no statistically significant differences among the groups for any parameter, as indicated by the high p-values (all above 0.3). This suggests that the groups are comparable across age, height, weight, and BMI as shown in Table 2.

**Table 2:** Demographic data among groups SD- Standard of deviation, BMI- Body Mass Index. p value <0.05 = statistically significant The p value is calculated using Analysis of Variance (ANOVA) test

Parameters	Group - A	Group - B	Group - C	P value
Age (Mean ±SD) years	25.6 ±3.8	25.5 ±3.8	25.7 ±4.4	0.974
Height (Mean ±SD) cms	161.9 ±3.8	162.1 ±4.1	161.4 ±3.7	0.7674
Weight (Mean ± SD) kg	67.26 ±10.10	64.53 ±7.77	64.7 ±11.98	0.5042
BMI (Mean ±SD) kg/m^2^	25.81 ±3.05	24.75 ±2.92	24.62 ±4.50	0.3741

It compares the pulse rate, systolic blood pressure (SBP), diastolic blood pressure (DBP), and mean arterial pressure (MAP) over time across three groups (A, B, C). Significant within-group differences are noted for pulse rate at eight minutes (p = 0.015), DBP at 10 and 12 minutes (p = 0.005, 0.004), and MAP at 10 and 12 minutes (p = 0.001, 0.003). Most other parameters did not show significant changes within groups over time as shown in Table 3.

**Table 3:** Hemodynamic parameters among groups SD- Standard of deviation, SBP- Systolic blood pressure, DBP- Diastolic blood pressure, MAP- Mean arterial pressure. p value <0.05 = statistical significant The p value is calculated using Repeated Measures Analysis of Variance (ANOVA) test

Parameters	Groups	Baseline	2 mins	4 mins	6 mins	8 mins	10 min	12 mins	15 mins
Pulse Rate (MEAN ±S.D)	Group-A	88.4 ±10.3	96.6 ±15.8	102.0 ±17.2	100.7 ±18.2	100.2 ±21.3	102.6 ±22.5	98.3 ±22.3	98.5 ±20.5
	Group-B	87.3 ±111.2	94.9 ±14.4	97.6 ±13.2	96.2 ±15.5	95.3 ±14.4	98.4 ±16.3	98.7 ±20.0	100.1 ±18.4
	Group-C	89.9 ±10.9	93.4 ±15.2	92.0 ±18.3	90.4 ±18.5	86.2 ±19.0	91.0 ±15.7	90.0 ±16.3	91.1 ±13.3
	P value (within group)	0.655	0.722	0.064	0.077	0.015*	0.052	0.160	0.117
SBP (MEAN ±S.D)	Group-A	121.8 ±10.9	115.7 ± 13.1	109.3 ±15.4	112.1 ±15.6	113.0 ±13.6	107.7 ±14.8	111.5 ±13.9	109.0 ±15.6
	Group-B	121.2 ±9.2	116.8 ±13.9	111.5 ±10.3	108.5 ±9.7	108.6 ±10.9	111.4 ±9.7	111.1 ±8.4	109.4 ±11.6
	Group-C	119.0 ±10.9	110.5 ±16.0	110.7 ±16.3	106.5 ±15.2	106.9 ±11.9	103.9 ±12.4	106.9 ±11.3	107.1 ±9.5
	P value (within group)	0.536	0.199	0.823	0.291	0.142	0.070	0.223	0.749
DBP (MEAN ±S.D)	Group-A	76.2 ±9.2	74.5 ±11.9	68.5 ±16.4	71.4 ±14.5	70.7 ±14.8	70.5 ±13.4	68.6 ±13.8	67.9 ±9.9
	Group-B	77.3 ±8.5	78.1 ±16.1	71.8 ±10.1	70.8 ±10.8	72 ±12.5	72.2 ±9.1	73.2 ±11.0	71.2 ±10.4
	Group-C	75.1 ±12.7	71.4 ±15.9	67.4 ±14.8	65.4 ±12.1	65.6 ±12.4	62.9 ±11.4	62.6 ±11.0	65.0 ±12.8
	P value (within group)	0.712	0.226	0.451	0.128	0.146	0.005**	0.004**	0.107
MAP (MEAN ±S.D)	Group-A	90.4 ±8.9	87.4 ±10.6	81.2 ±15.2	84.9 ±13.5	84.4 ±13.5	82.3 ±12.6	82.4 ±13.9	81.1 ±11.1
	Group-B	91.5 ±7.7	90.5 ±14.4	84.7 ±9.1	82.8 ±9.9	83.8 ±11.2	84.8 ±7.9	85.5 ±9.3	83.7 ±10.4
	Group-C	89.3 ±11.8	83.5 ±14.7	80.2 ±13.4	77.9 ±11.6	77.6 ±11.5	74.8 ±9.3	75.7 ±9.3	77.8 ±10.5
	P value (within group)	0.660	0.131	0.368	0.067	0.060	0.001**	0.003**	0.110

It summarizes the incidence of a 20% fall in systolic blood pressure across three groups (A, B, and C). Group C had a slightly higher percentage (60%) of participants experiencing this fall compared to Group A (46.7%) and Group B (46.7%). The overall comparison shows no statistically significant difference between the groups (ꭓ² = 1.423, p = 0.491) as shown in Table 4.

**Table 4:** Comparison of number of patients showing 20% fall of systolic blood pressure between the groups. The p value is calculated using Chi-square test

Systolic Blood Pressure			Groups			Total	ꭓ 2 - value	p-value
			Group A	Group B	Group C			
20% fall	Yes	Count	14	14	18	46	1.423	0.491 #
		%	46.7%	46.7%	60.0%	51.1%		
	No	Count	16	16	12	44		
		%	53.3%	53.3%	40.0%	48.9%		
Total		Count	30	30	30	90		
		%	100.0%	100.0%	100.0%	100.0%		
# No Statistical Significance at p > 0.05 level								

It presents the number of participants in each group (A, B, and C) who required ephedrine. Group C had the highest percentage needing ephedrine (66.67%), followed by Group A (53.33%) and Group B (46.67%). The difference among the groups is not statistically significant (χ² = 2.52, df = 2, p = 0.28) as shown in Table 5.

**Table 5:** Comparison of Patients required Ephedrine among groups P Value <0.05 is Considered Significant The p value is calculated using Chi-square test

Group	Ephedrine Required		Total	Significance
	Yes	No		
Group A	16 (53.33)	14 (46.67)	30 (100)	Χ^2 ^= 2.52, df= 2, P value= 0.28
Group B	14 (46.67)	16(53.33)	30 (100)	
Group C	20 (66.67)	10(33.33)	30 (100)	

It shows the number of patients requiring ephedrine and the total dose needed across three groups. Group C had the highest number of patients (20) and required the most ephedrine (174 mg), followed by Group A with 16 patients needing 126 mg, and Group B with 14 patients requiring 90 mg. This suggests a higher demand for ephedrine in Group C as shown in Table 6.

**Table 6:** Total dose of ephedrine required to treat hypotension among groups.

	Group-A	Group-B	Group-C
Number of patients required ephedrine dose	16	14	20
Total dose of ephedrine required (mg)	126mg	90mg	174mg

It compares the mean and median values for Groups A and C. Group A had a mean of 7.88 (SD 2.87) and a median of 6 (IQR 6-12), while Group C had a mean of 8.7 (SD 3.63) and a median of 6 (IQR 6-12). The difference between the groups is not statistically significant (U = 143.50, p = 0.5425) as shown in Table 7.

**Table 7:** Table showing comparison of dose of ephedrine required among Group A and Group C The p value is calculated using Mann-Whitney U test

Group	Mean (SD)	Median (IQR)	Significance
Group A	7.8750 (2.8723)	6 (6-12)	U= 143.50, P value = 0.5425*
Group C	8.7000 (3.6288)	6 (6-12)	

It compares Groups B and C on their mean and median values. Group B had a mean of 6.43 (SD 1.60) and a median of 6 (IQR 6-6), while Group C had a mean of 8.7 (SD 3.63) and a median of 6 (IQR 6-12). The difference between the groups is statistically significant (U = 93.50, p = 0.0420) as shown in Table 8.

**Table 8:** Table showing comparison of dose of ephedrine required among Group-B and Group-C The p value is calculated using Mann-Whitney U test

Group	Mean (SD)	Median (IQR)	Significance
Group B	6.4286 (1.6036)	6 (6-6)	U= 93.50, P= 0.0420
Group C	8.7000 (3.6288)	6 (6-12)	

It summarizes the complications experienced by participants across three groups (A, B, and C), including nausea, shivering, vomiting, and combinations thereof. The most common complication was shivering, occurring in 25.6% of participants, while 42.2% experienced no complications. There is no statistically significant difference in the incidence of complications among the groups (χ² = 6.890, p = 0.865) as shown in Table 9.

**Table 9:** Comparison of Complications between Groups The p value is calculated using chi-square test.

Complications			Groups			Total	ꭓ 2 - value	p-value
			Group A	Group B	Group C			
Complications	Nausea	Count	2	6	5	13	6.890	0.865 #
		%	6.7%	20.0%	16.7%	14.4%		
	Shivering	Count	8	7	8	23		
		%	26.7%	23.3%	26.7%	25.6%		
	Vomiting	Count	3	2	3	8		
		%	10.0%	6.7%	10.0%	8.9%		
	Nausea, Shivering	Count	1	2	1	4		
		%	3.3%	6.7%	3.3%	4.4%		
	Vomiting , Shivering	Count	1	0	0	1		
		%	3.3%	0.0%	0.0%	1.1%		
	All three	Count	2	0	1	3		
		%	6.7%	0.0%	3.3%	3.3%		
	No complications	Count	13	13	12	38		
		%	43.3%	43.3%	40.0%	42.2%		
Total		Count	30	30	30	90		
		%	100.0%	100.0%	100.0%	100.0%		
# No Statistical Significance at p > 0.05 level								

## Discussion

In our study we found that Group B showed significant decrease in ephedrine requirement compared to Group C (6.4286mg±1.6036 vs 8.7mg±3.6288, p=0.0420). Prophylactic use of IV glycopyrrolate plus IV ondansetron (Group A) and IM glycopyrrolate plus IV ondansetron (Group B) did not result in significant reduction in incidence of post-spinal hypotension during LSCS (p=0.491). Our finding is similar to findings by Deshar et al. [21]. Similar to our findings, a study by Manem et al. showed that requirement of vasopressor is less with IM glycopyrrolate [19].

The difference in ephedrine requirement in Group A was not statistically significant when compared to Group C (7.8750mg±2.8723 vs 8.7mg±3.6288, p=0.5425). Group C (IV ondansetron only) did not reduce incidence of hypotension and vasopressor requirement. We have given injection ondansetron in the control group because it is the institutional protocol to give preoperatively IV ondansetron. Similar to our finding Samarah et al. [22] in their study concluded that with IV ondansetron there was no reduction in incidence of post-spinal hypotension in LSCS but there was decrease in vasopressor requirement. Contrary to our finding, Sahoo et al. concluded that decrease in systolic blood pressure was reduced in IV ondansetron group [23]. Difference in the findings may be because they have not defined the definition of hypotension and vasopressor was given when systolic blood pressure was less than 90mmHg, they have also not considered total dose of vasopressor required to treat hypotension.

Group C showed significantly more fall in MAP (74.8±9.3) as compared to Group A (82.3±12.6, p=0.014) and Group B (84.8±7.9, p=0.001) at 10th minute. At the 12th minute fall in MAP in Group C (75.7±9.3) was statistically significant compared to Group B (85.5±9.3 p=0.002) and statistically insignificant when compared to Group A (82.4±13.9, p=0.052). MAP after fall was in clinical range so it was not clinically significant. Group A showed increase in heart rate at eight minutes compared to Group B and Group C (100.2±21.3 vs 95.3±14.4 vs 86.2±19.0, p=0.015).

A study by Manem et al., with comparison of IM glycopyrrolate and IM normal saline showed lesser incidence of post-spinal hypotension with IM glycopyrrolate [19]. A study by Rucklidge et al. [24] found that severity of hypotension was similar in both the groups treated with IV glycopyrrolate and normal saline. They assessed severity of hypotension by requirements of ephedrine boluses. Vandhan et al. showed that combination of IV glycopyrrolate, ondansetron and ephedrine have a better haemodynamic stability in terms of total vasopressor consumption and incidence and severity of hypotension in comparison to on-demand ephedrine boluses during obstetric spinal anaesthesia [20].

Findings about prevention of hypotension are similar to the meta-analysis conducted by Patel et al. where prophylactic intravenous glycopyrrolate during spinal anaesthesia for caesarean delivery does not prevent hypotension while increasing maternal heart rate [18]. However contrary to our study, requirement of vasopressor is reduced in their study with IV glycopyrrolate. One of the reasons for this conflicting result is the variation in doses and timing of glycopyrrolate administration, which may affect the outcome measures. Two studies used 4mcg/kg of glycopyrrolate [25,26], two studies used 0.2mg [27,28] and one study used 0.4mg [29]. Timing of administration of glycopyrroalte was also different: 25 minutes before spinal anaesthesia [29], as spinal anaesthetic given [26], during fluid preload [27], immediately before spinal anaesthesia [25] and immediately after spinal anaesthesia [28]. Whereas a study by Prasad found that IV glycopyrrolate showed significant decrease in vasopressor requirement and significant difference in incidence of hypotension [30], this contrary finding may be because they did not define the definition of hypotension and phenylephrine was given in continuous infusion of 50mcg/min which might have affected the outcome.

In our study, no difference was detected in incidence of intraoperative nausea, vomiting and shivering between the groups. There are different studies with different results about intraoperative nausea and vomiting. Two studies showed a reduction in incidence of nausea and vomiting with the use of prophylactic use of glycopyrrolate [18,25]. And in these studies, prophylactic vasopressors were not used in preventing hypotension. Whereas when prophylactic vasopressors were used pre-treatment with glycopyrrolate did not offer any advantage in terms of intra-operative nausea and vomiting (IONV) [25,26]. Intraoperative nausea and vomiting during spinal anaesthesia for caesarean section has multiple contributing factors like hypotension, vagal hyperactivity, rescue IV opiods, uterotonic agents, visceral pain, etc. so it is difficult to find out the relationship between prophylactic glycopyrrolate and its effect on IONV.

The limitations of the study are to measure blood pressure, conventional non-invasive monitors were used which might be less accurate than intra arterial pressure. As the size of the uterus varies and there is variation in pulse and blood pressure among the patient during caesarean section, study should be done on more patients. As duration of labour varies which affects hemodynamic parameters study should be done separately on elective and emergency LSCS. Pre-op intravascular fluid assessment was not done. Future studies are required along with preop intravascular fluid assessment to avoid confounding effects of fluid deficit. 

## Conclusions

In conclusion, this study evaluated the efficacy of IV glycopyrrolate combined with IV ondansetron versus IM glycopyrrolate combined with IV ondansetron in reducing post-spinal hypotension during cesarean sections. While the IM glycopyrrolate group (Group B) demonstrated a significant reduction in the total ephedrine dose required to manage hypotension compared to the control group, there was no significant reduction in the incidence of hypotension across the groups. The IV glycopyrrolate group (Group A) showed an increase in maternal heart rate but no significant impact on vasopressor requirements. Moreover, no significant differences were observed in the occurrence of intraoperative complications such as nausea, vomiting, or shivering among the groups. Our findings suggest that while IM glycopyrrolate reduces the need for vasopressors, neither IV nor IM glycopyrrolate significantly prevents post-spinal hypotension. This study underscores the complexity of managing hypotension in cesarean sections and highlights the need for further research to optimize prophylactic strategies.
